# Antigen-Specific Immune Tolerance in Multiple Sclerosis—Promising Approaches and How to Bring Them to Patients

**DOI:** 10.3389/fimmu.2021.640935

**Published:** 2021-03-22

**Authors:** Andreas Lutterotti, Helen Hayward-Koennecke, Mireia Sospedra, Roland Martin

**Affiliations:** Neuroimmunology and MS Research Section, Neurology Clinic, University Hospital Zurich & University of Zurich, Zurich, Switzerland

**Keywords:** multiple sclerosis, immune tolerance, therapy, antigen-specific, target antigens

## Abstract

Antigen-specific tolerance induction aims at treating multiple sclerosis (MS) at the root of its pathogenesis and has the prospect of personalization. Several promising tolerization approaches using different technologies and modes of action have already advanced to clinical testing. The prerequisites for successful tolerance induction include the knowledge of target antigens, core pathomechanisms, and how to pursue a clinical development path that is distinct from conventional drug development. Key aspects including patient selection, outcome measures, demonstrating the mechanisms of action as well as the positioning in the rapidly growing spectrum of MS treatments have to be considered to bring this therapy to patients.

## Introduction

Therapeutic interventions in medicine should provide the highest possible specificity and well-known mechanisms when targeting the pathogenic processes underlying a specific disease. In the evolving era of precision medicine, this aspect has become the most important goal of treatment development and is driven by advances in the understanding of disease etiology and relevant pathomechanisms. Immune-mediated diseases, including autoimmune disease (AIDs) and allergies, which together affect up to 20% of the population in industrialized countries, are important examples, in which the field aims to move from broadly immunomodulatory to highly specific treatments.

Organ-specific AIDs are characterized by acute or chronic inflammation driven by an autoreactive immune response against self-antigens. Although the relative contribution of different cellular and humoral immune effector mechanisms differ between diseases and even individual patients, the selective abrogation of the autoreactive immune response offers the opportunity to specifically treat and potentially cure an AID (1). The concept of reverting autoimmunity by induction of antigen-specific immune tolerance stands in contrast to currently available therapies, which target the inflammatory immune response broadly, often compromise protection against infections and may even lead to secondary autoimmunity. This is particularly relevant in chronic diseases affecting young patients with consequential need for long-term immunosuppression. One such example is multiple sclerosis (MS).

MS is considered a prototypic, organ-specific AID characterized by chronic inflammation of the brain and spinal cord leading to variable neurologic signs and symptoms and often persisting disability (2). Although the development of effective therapies has been very successful over more than two decades, it has come at the cost of sometimes severe safety concerns related to the lack of specificity and global immunosuppression.

Approaching therapeutic immune tolerance in MS requires a sound understanding of its autoimmune pathogenesis including the main target antigens as well as the mechanisms of immune tolerance and suitable methods to assess the effects of a tolerization regimen. The clinical development of tolerization poses several challenges, which are related to the disease itself, the mechanisms of the tolerizing approach and clinical trial design, which all need to be mastered for successfully advancing tolerization to the clinic.

Here, we will provide an overview about the current state of knowledge of target antigens and immune tolerance mechanisms in MS, discuss lessons learned from previous attempts towards tolerization and what we consider the main hurdles during clinical development of antigen-specific therapies (ASTs). While several innovative tolerization approaches are currently in pre-clinical development [reviewed in (3)], we will focus only on those that have already entered clinical phases.

## Target Antigens in MS—Old Candidates and New Developments

One core prerequisite for developing antigen-specific tolerization is the firm knowledge of the relevant target autoantigens. Although this aspect has received a lot of attention in the past, the antigen specificity of autoreactive T cells and also of autoantibodies in MS has been examined only by a few groups during recent years comprehensive reviews in (1, 4–8). Since demyelination is one hallmark of MS lesions, the search for targets focused initially on myelin proteins such as myelin basic protein (MBP), proteolipid protein (PLP) and myelin oligodendrocyte glycoprotein (MOG), which had been shown to be encephalitogenic in the animal model experimental autoimmune encephalomyelitis (EAE) (7, 8). Several of the encephalitogenic peptides of MBP, PLP and MOG are also immunodominant in MS patients (4, 7, 9), and peptides of other myelin- (2’-3’ cyclic nucleotide phosphodiesterase (CNPase), myelin oligodendrocyte basic protein (MOBP), oligodendrocyte-specific protein (OSP), myelin-associated glycoprotein (MAG)) and non-myelin proteins (alpha-B crystallin, transaldolase H, S100 beta, contactin 2/TAG-1, RAS guanyl-releasing protein 2, RASGRP2, GDP L-fucose synthase, TSTA3, KIR4.1, anoctamin 2) have been described (5, 6, 10–12), but not yet studied to the same extent.

To assess the biological relevance of putative target autoantigens, the criteria outlined in **Box 1** can be used, which we have weighed based on current knowledge (see also **Table 1**). The selection of target antigens is based on the consideration that autoreactive and proinflammatory CD4+ T cells that are restricted by MS-associated HLA-DR molecules are the drivers of the disease, and hence that antigen-specific tolerization should silence/eliminate these cells (4, 7). **Table 1** summarizes the antigens that have been identified and which properties support their importance. We consider the following autoantigens most important in the moment due to the fact that: (i) they are targets of high avidity autoreactive T cells in MS [MBP13-32; MBP111-129, MBP146-170; PLP139-154, MOG1-20 and MOG35-55 (14)], (ii) their encephalitogenicity has been shown with humanized mice expressing T cell receptors (TCRs) of MS patient-derived T cell clones (MBP 83-99), or (iii) because they have been shown to be targeted by brain- and CSF-infiltrating T cells of MS patients (GDP L-fucose synthase, TSTA3, and RASGRP2) (11, 12) (for details see **Box 1** and **Table 1**). Reactivity against the above mix of high avidity myelin peptide targets and MBP 83-99 has been examined in MS patients in North America (14), Germany (15), Spain (16) and Switzerland (own unpublished data), and between 74% and 100% of patients have shown reactivity. This cocktail is a good start since the majority of patients reacts to one or more peptides, and it has been used by us (15) and also adopted by other groups (17) for tolerization trials. It is not clear in the moment, how many peptides are ideal, but we assume that including as many relevant target antigens as possible will increase the likelihood of successful tolerization, particularly if the disease is already ongoing for longer time, and hence it is likely that the autoimmune response is directed against multiple peptides of one (intramolecular) or several proteins (intermolecular), known as epitope spreading (18–20). In this context, the ability of the tolerization approach to prevent epitope spreading is crucial. The use of myelin peptide-coupled splenocytes has been very effective in that respect (21), but to our knowledge prevention of epitope spreading are less well or not examined and shown for other tolerization modalities (22).

Box 1 **Criteria to judge the relevance of target autoantigens (key criteria in bold).**

- **Recognized by CSF- and/or brain-infiltrating T cells; recognized by autoproliferating T cell fraction**
- **Use of the respective peptide or protein or derivative thereof has shown tolerizing activity in tolerance trial in MS**
- **Immunodominant for (proinflammatory) CD4+ T cells in MS patients in the context of one or several MS-associated HLA-DR molecules**
- **Recognized with high(er) avidity by T cells of MS patients**
- **Encephalitogenic in EAE models**
- **MS patient-derived TCR with specificity for the antigen is encephalitogenic in humanized mouse models; or encephalitogenic in HLA-DR humanized tg mice**
- “Encephalitogenic” in MS patients*
- T cell cross-reactivity between autoantigen and MS-associated pathogen/s, e.g. EBV, *Akkermansia*
- Autoantibody cross-reactivity between autoantigen and MS-associated pathogen/s, e.g. EBV, *Akkermansia*
- Target of autoantibodies in MS and pathogenicity shown in EAE:
- Exclusive expression in the brain (relative)
- Generation of peptide is independent of antigen processing and mimics naturally occurring sequences

* Refers to the special case of increased disease activity following vaccination with an altered peptide ligand of MBP 83-99 (13).

**Table 1:** Autoreactive T cell targets in multiple sclerosis: evidence for relevance.

Protein	Peptide	Recognized by brain- and/or CSF-infiltrating T cells	Tolerizing activity in humans	Immuno-dominant in MS patients	High avidity recognition	Encephalitogenic (EAE)	Encephalitogenic in humanized models	References(see supplement for detailed list)
**MBP**	**Protein**							S*1,S2*
	Ac 1-9							S*3-S7*
	**13-31**							S*3-S8*
	30-44 (p.i.)							S*9, S10*
	69-86							S*4*
	79-87							S*4, S11, S12*
	83-99* (p.i.)							S*9, S13-S19*
	96-109							S*4*
	110-118							S*20*
	**111-129**							S*8, S21, S22*
	130-144 (p.i.)							S*9, S10*
	140-154 (p.i.)							S*9, S10*
	**146-170**							S*9, S23*
**PLP**	**Protein**							S*24, S25*
	40-60						*	S*26-S29*
	56-70							S*27, S30*
	89-106							S*31-S34*
	95-117							S*31*
	**139-154**							S*35, S36*
	178-197							S*26, S37*
	**190-208**							S*25*
	**184-209**							S*25, S37*
	**217-233**							S*38*
MOG**	**Protein**							S*39-S45*
	**1-20**							S*46-S48*
	11-30							S*47*
	21-40							S*20, S49*
	31-50							S*20, S49*
	**35-55**							S*8, S46, S49-S51*
	63-87							S*47, S48*
	64-96							S*46, S49, S52-S56*
	97-108							S*52-S56*
	119-132							S*58, S59*
	146-154							*S56, S58*
	181-195							*S58, S59*
	186-200							*S58, S59*
MOBP	15-36							S*60-S62*
	21-39					***		S*60, S63*
	37-60							S*63*
	65-86/55-77							S*61, S64*
CNPase	**Protein**							S*60, S65*
	343-373							S*66*
	356-388							S*66-68*
MAG								S*65, S69-S75*
OSP/claudin 11								S*65, S76-S81*
TSTA3****	51-65							S*82* Own unpublished data
	136-150							S*82* Own unpublished data
	242-251							S*82* Own unpublished data
	296-310							S*82* Own unpublished data
RASGRP2****	78-87							S*83* Own unpublished data
Transaldolase H								S*84-S87*
α-B Crystallin								S*88-S95*
Neurofascin *****								S*96-S98*
Contactin-2/TAG-1								S*98-S100*

green color indicates that the respective evidence has been shown, red color that it has been tested and was negative. White = not known and/or not done. For some peptides, independence of antigen processing has been documented. If this was the case, it is indicated after the amino acid number by “(p.i.)”. The table only mentions T cell antigens. Antibody targets, e.g. KIR4-1 or anoctamin-2, have been omitted. MBP, myelin basic protein; PLP, proteolipid protein; MOG, myelin oligodendrocyte glycoprotein; MOBP, myelin associated oligodendrocyte basic protein; CNPase, 2’,3’-Cyclic-nucleotide 3’-phosphodiesterase; MAG, myelin-associated glycoprotein; OSP, oligodendrocyte-specific protein; RASGRP2, Ras-guanyl releasing protein 2; TSTA3, GDP L-fucose synthase.

*Humanized TCR and A*03:01 transgenic mouse.

**Exclusive expression in the CNS debated (Pagany et al. Neurosci. Lett. 2003).

***Encephalitogenic epitope in EAE different.

****Immunodominant epitopes in part mapped, but studies ongoing.

*****Anti-neurofascin antibodies lead to axonal damage when combined with co-transfer of myelin-specific T cells.

Extensive additional information on the topic can be found reviews from group (S2, S20).

An additional criterion to select peptides for tolerization is their independence of antigen processing. During antigen processing, proteins are digested by specific proteases, and it has been demonstrated that peptides that are generated by the naturally occurring processing mechanisms are protected from degradation (23, 24) and that this aspect is relevant for tolerance induction. Peptides MBP30-44, MBP83-99, MBP131-145 and MBP140-154 fulfill these criteria and have been tested clinically (see below).

Regarding targets of autoantibodies identified in MS such as KIR4.1 (25) and anoctamin 2 (26), it will be important to examine whether these autoantigens are also recognized by autoreactive CD4+ T cells and if further evidence supports their pathogenetic relevance before including them in tolerization trials.

In summary, careful examination of disease-relevant target antigens, which shall be used for tolerance induction, is warranted. Based on the criteria outlined in **Box 1** and **Table 1**, we will soon add immunodominant peptides derived from TSTA3 (12) and RASGRP2 (11) to the tolerizing cocktail of high-avidity myelin peptides that we currently use for tolerization.

## Mechanisms of Immune Tolerance—Knowledge in Animal Models and Humans

Unresponsiveness of the adaptive immune system against self-antigens is generated by so-called central tolerance mechanisms in the thymus for T cells and in the bone marrow for B cells. Central tolerance assures that T cells that recognize self-antigens with high avidity are eliminated by apoptosis, a process called negative selection, while T cells that respond with low avidity (that is only at high antigen concentration) are positively selected into the peripheral immune system to protect the host from pathogens. This mechanism destroys potentially dangerous T cells with specificity for most self-antigens, however, it also implies that all peripheral blood T cells are able to recognize autoantigens and are to some extent autoreactive. Peripheral tolerance mechanisms including anergy, a state of functional silence when T cells are stimulated in the absence of costimulatory molecules, non-responsiveness at low antigen concentrations and the deletion of autoreactive T cells by activation-induced cell death (AICD) assure under physiological conditions that pathologic autoreactivity is avoided [reviewed in (27)]. The latter mechanisms are antigen-specific, but not expected to mediate long-lasting non-responsiveness. Further control mechanisms include several types of T regulatory cells (Tregs), most notably natural, thymus-derived Tregs (nTregs), which are characterized among other markers by expression of the transcription factor FoxP3 (28), and so-called induced, IL-10-secreting Tregs (iTregs or Tr1 cells) (29). In the context of therapeutic tolerance induction, the activation and expansion of Tregs is critical for actively controlling autoreactive T cells against multiple antigens. A phase Ib/IIa using autologous Tregs in MS patients has been reported recently with good safety results (30). Different from the above elimination of autoreactive T cells by apoptosis or silencing by anergy, Treg-mediated tolerance is expected to last long(er) and be able to control a broader range of autoreactive T cells. An important aspect that has not been addressed well in humans/MS is, to what extent Tregs need to be antigen-specific. Finally, there are various other immunoregulatory cell populations including B regulatory cells (31), regulatory plasma cells (32), CD56^bright^ natural killer cells (33) and others, which will not be addressed here.

Many modalities to induce immune tolerance have been tested with varying success in animal models (3). These include different routes of administration (RoA) of autoantigens, for instance oral, nasal (generally mucosal), transdermal or intravenous application, coupling of autoantigens (usually peptides) to cells (white blood cells, red blood cells) or other carriers like nanoparticles, but also the intramuscular injection of a plasmid encoding MBP for ectopic expression in muscle. The experimental data, mechanism/s of action and potential caveats have been reviewed (3). Furthermore, not only the RoA, but also the site of degradation of tolerizing peptides and the context of their presentation to the immune system, that is tolerogenic or immunogenic/inflammatory, are critical. The generation and maintenance of peripheral tolerance against proteins that enter the body *via* the gut or the natural degradation of dying cells in the body occur preferentially in the liver and spleen, while antigen processing and presentation in lymph nodes or in an inflammatory context induce proinflammatory immune responses instead.

For certain tolerization approaches, for instance peptide-coupled fixed white or red blood cells and antigen-coated nanoparticles, the mechanistic data are robust and both prophylactic and long-lasting therapeutic effects have been shown (21, 22). Fixed, peptide-coupled cells induce tolerance by several mechanisms including anergy and the expansion of Tregs, and further they block epitope spreading (34).

The translation of such a therapy to patients poses multiple challenges, particularly to demonstrate that autoreactive T cells are silenced and/or deleted and that the approach is indeed antigen-specific. Different from anergy induction, which will require repeated administration of autoantigen over long/er periods of time, it is desirable and needs to be shown that active peripheral tolerance mechanisms, particularly the induction/expansion of Tregs can be achieved. Ideally, the mechanistic studies that accompany the clinical trials should be able to demonstrate that the putative mechanism(s) of action of the respective approach indeed operate in patients.

The main difficulties are outlined in the following. As described above, autoreactive T cells express low avidity TCRs and are also present in healthy donors (14, 35, 36). Distinguishing pathogenic autoreactive T cells from the “physiological” level of autoreactivity is therefore very difficult. The functional phenotype of CD4+ T cells, which in the case of MS are mainly Th1 and Th1* cells based on certain chemokine receptor profiles, expression of signature transcription factors or cytokines like IFN-γ and IL-17, can in principle be used, but also are not easy to quantitate reliably. Further, pathogenic autoreactive T cells are rare. Depending on the assay that is used, frequencies range between a few percent to 1 in 10^7^ (37, 38). It is therefore difficult to reliably enumerate autoreactive CD4+ T cells with a certain specificity before therapy, but even more to show their reduction or change of phenotype after tolerization. Testing sufficient numbers of cells *in vitro* and to use a sensitive assay are both important. We have recently employed a protocol modified from Geiger et al. (39), primary proliferation with peripheral blood T cells without pre-selection (15), and a Fluorospot assay with bead-coupled whole myelin proteins (40) to successfully quantify these cells (41). Equally demanding and currently not solved are methods to reliably identify and enumerate the different Treg populations, most importantly nTregs and Tr1 cells. Again, their low frequency is one problem. Further, surface markers of nTregs, CD25 and CD39, are not specific for these cells (42). Intracellular detection of FoxP3 is more demanding and, in order to demonstrate functionally stable Treg differentiation, the methylation status of the FoxP3 locus is better, but not established for easy detection of nTreg numbers. Accurate enumeration of Tr1 cells (CD3+, CD4+, CD45RA-, CD49b+, LAG3+) by flow cytometry (43), is difficult, again due to their low numbers. IL-10, their signature cytokine, may be used as a surrogate for Tr1 function, however, IL-10 is not exclusively produced by Tr1 cells, and serum levels are at the limit for detection. Finally, biomarkers that are related to damage of the target tissue (for example neurofilament light chain) may be used as an indirect measure for a tolerizing effect if their levels drop after treatment (44).

In summary, the mechanistic testing should demonstrate immunosafety, that is the absence of a vaccination response that induces rather than abrogates autoimmune inflammation as most important acute safety concern. With respect to proving the mechanism(s) of action (MoA), the accompanying *in vitro* testing should query putative peripheral tolerance effects including the reduction or elimination of pathogenic, autoantigen-specific T cells, the induction of regulatory T cells and their cytokines as well as markers that indicate the reduction of inflammation and damage in the target organ (see **Box 2**). Testing of CSF parameters is highly desirable since they likely better reflect pathogenic immune mechanisms in the target organ, but not possible in larger clinical trials. Besides establishing the MoA of the tolerizing regime and providing indications for its immunological efficacy, these studies are important for finding the best dose and dosing interval. Successful development of tolerization therapies will depend on whether the above described challenges of mechanistic studies can be overcome or not.

Box 2 **Goals and assays for testing the mechanism/s of action of tolerance induction.**

**To assess immunosafety and exclude that the respective approach induces disease activity, loss or increase of immune cells**

-Standard hematology and flow cytometry testing (or mass cytometry) for the major populations (CD4+ and CD8+ T cells, B cells, monocytes, dendritic cells, NK cells, NK-T cells)

**Assess the loss/decrease of antigen-specific autoreactive T cells and change of phenotype**

-Various types of proliferation assays using sufficient numbers of input cells

-Intracellular cytokine staining, chemokine receptor profiles by surface staining

-Combination of the above can be achieved by Fluorospot testing assessing numbers of antigen-specific T cells and their functional phenotype

-Antigen/HLA-DR tetramers for direct enumeration of autoreactive cells

**Induction of T regulatory cells**

-Flow cytometry testing for nTregs and Tr1 cells

-Support nTreg induction by demonstrating demethylation status of Fox-P3 (quantitative PCR)

-Support Tr1 cell increase by intracellular cytokine staining and/or increase of serum IL-10

-Functional assays

**Biomarkers indicating reduced target organ damage and/or reduction of inflammation**

-Markers for neuronal/axonal damage or brain inflammation, for example neurofilament light chain (NFL)

## Approaches to Immune Tolerance and Lessons Learned

**Figure 1:**
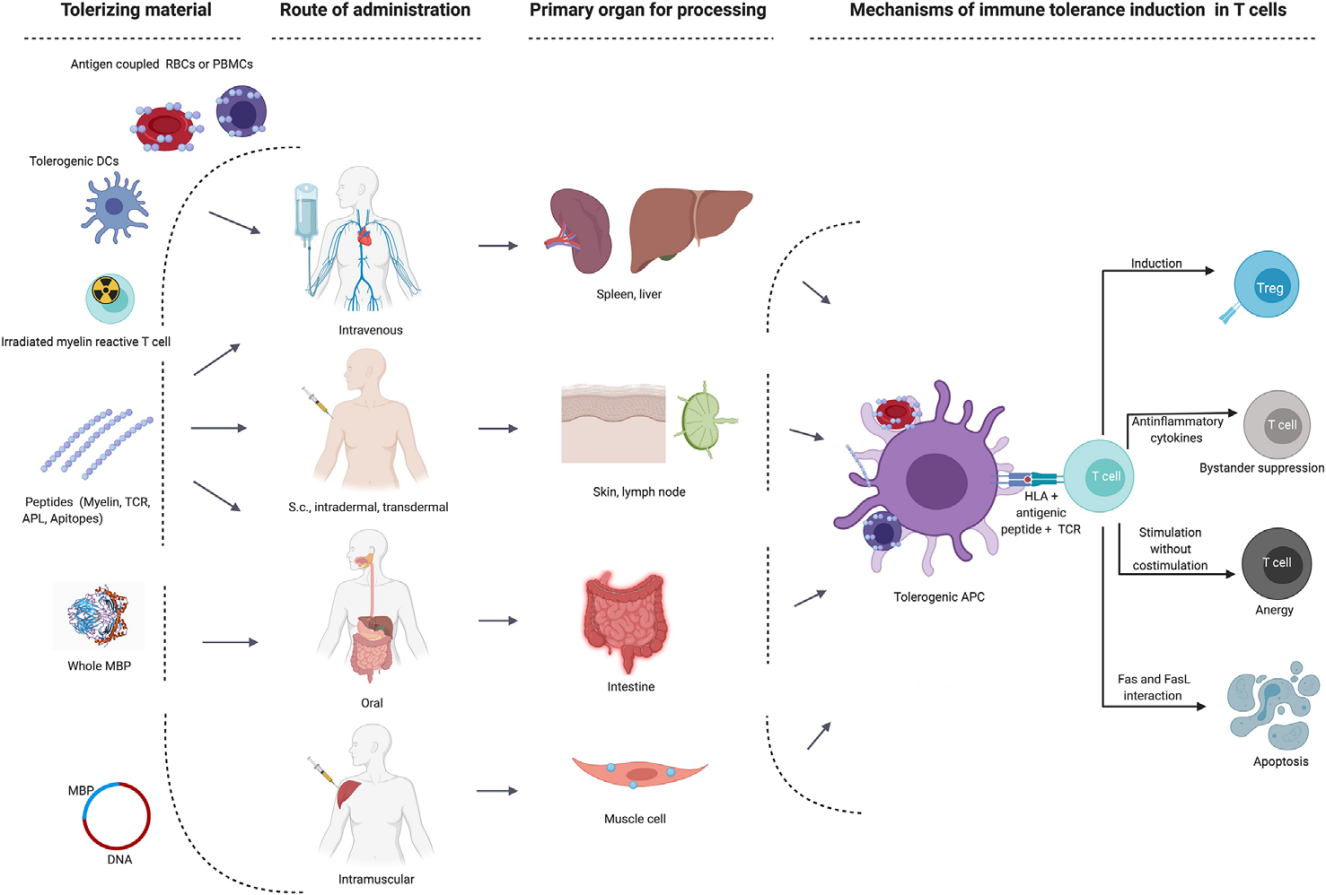
Main target organs and mechanisms of action depending on route of administration in different immune tolerance strategies. APC, antigen-presenting cells; DC, dendritic cell; MBP, myelin basic protein; RBCs, red blood cells; s.c., subcutaneous; TCR, T cell receptor; Treg, regulatory T cell.

The appeal of selectively silencing the autoimmune response without impairing protective immunity has prompted numerous efforts to translate promising results of ASTs from animal models to the clinic. ASTs employed different approaches ranging from the use of whole proteins, peptides in various routes of administration, tolerogenic dendritic cells, DNA, T cell or TCR vaccinations, all operating *via* different mechanisms and most of them targeting the trimolecular complex between HLA-class II molecule, antigenic peptide and a CD4+ T cell’s TCR (**Figure 1** and **Table 2**). While most of the early tolerization trials failed to reach their clinical endpoints despite promising mechanistic results, some of the recent studies have been encouraging in affecting imaging-based outcomes in early phase clinical trials. Below, we will summarize the most important observations and lessons from tolerance trials in MS. A detailed list of all trials and their main characteristics and findings is given in **Table 2**.

**Table 2:** Antigen-specific tolerization approaches in the clinic.

DNA vaccination										
Ref. & year	Substance	# pat./MS type	Route of admin.	Dose/Frequency	Study phase	Study design	Trial duration	Outcome parameters	Safety/clinical/immunological parameters
(45) 2007	BHT-3009 DNA vaccine encoding full length human MBP combined with atorvastatin calcium	30 (11 RRMS, 19 SPMS)	i.m.	0,5mg, 1,5mg or 3mg at weeks 1,2,5 and 9	Phase 1/2	Randomized, placebo-controlled, double blind dose escalation study	13 w, then unblinded, follow up 50 w	**Primary endpoint:** safety **Others:** immune response as measured by T-cell activity in CSF	**Safety results:** safe and well tolerated **Clinical:** trend toward decrease of GD-enhancing lesions on MRI **Immunological:** antigen specific downregulation of autoimmune activity in blood and CSF, decline of myelin-reactive, IFNγ-producing CD4+ T cells **Other**: no beneficial effect of atorvastatin
(46) 2008	BHT-3009 DNA vaccine encoding full length human MBP	289 RRMS	i.m.	1.5mg or 0,5mg at weeks 0,2,4, then every 4 weeks until week 44	Phase 2	Randomized placebo-controlled trial	48 w	**Primary endpoint**: 4 week rate of occurrence of new Gd-enhancing lesions on MRI from weeks 28 to 48 **Secondary:** total number and volume of new Gd-enhancing lesions	**Safety results:** safe and well tolerated **Clinical:** lower dose led to a decrease in Gd lesions, no beneficial effect on disease course. Higher dose ineffective **Immunological:** lower dose was associated with significant decrease of autoantibody titers
**Peptide- and protein-based approaches**										
**Ref. & year**	**Substance**	**# pat./MS type**	**Route of** **admin.**	**Dose/Frequency**	**Study phase**	**Study design**	**Trial duration**	**Outcome parameters**	**Results**
(47) 1993 *	Bovine myelin	30 early RRMS	oral	300mg daily for 1 year	Phase 1	Double blind, randomized for age, disease duration, EDSS, number of exacerbations in previous 2y	1y	**Primary endpoint:** number of major exacerbations, change in disability as measured on EDSS	**Safety:** no toxicity **Clinical:** overall change in EDSS in myelin not greater than in placebo **Immunological:** no increase of proliferation to MBP and PLP in treated patients, overall frequency of MBP reactive T cells in oral myelin treated group decreased
(48) 1994 *	TCR peptide vaccine (Vβ5.2 and Vβ 6.1)	11 progressive MS	I.d.	Initially: 4 weekly injections of 100µg, then incremental doses every 4 weeks: 100, 200, 300, 600, 1500 and 3000µg; after dose escalation patients were started on second peptide with or without first peptide being continued	Phase 1	Open label	No information	Assessment of immunogenicity and safety	**Clinical:** one patient improved, 4 stable, 2 worsened among peptide responders **Immunological:** low dose (100 to 300µg) induced T cell immunity. Delayed type hypersensitivity skin responses in 3 patients; generation of TCR peptide-specific ab in two patients
(49) 1996 *	TCR peptide vaccine (Vβ5.2 sequence)	23 (8 PPMS; 15 SPMS) (HLA-DRβ1*1501^+^)	i.d.	100µg weekly for 4 weeks, then monthly for 10 mo (in total 14 injections)	Phase 1	Double blind, placebo-controlled	12mo	Clinical parameters, TCR peptide immunogenicity, effects on MBP response	**Clinical:** no clinical progression in TCR responders **Immunological:** boosting of T cell responses to Vβ5.2, reduced frequency of MBP-specific T cells
(50) 1997 *	TCR peptide vaccine (Vβ6 CDR2 peptide)	10 MS	i.m.	5 patients: 100µg 2x in 4 weeks. 5 patients: 300µg 2x in 4 weeks	Phase 1	Open label	24 w	Assessment of toxicity, immunogenicity and biological effects in CSF	**Overall:** safe (no SAEs) **Clinical:** no significant changes in physical examination, disability score stable, no increase in new MRI lesion load **Immunological:** anti-peptide ab not detectable, in high dose group: marked decrease of Vβ6 T cells and minor decrease in CSF cellularity
(51) 2005	Trivalent TCR BV5S2, BV6S5 and BV13S1 CDR2 peptides with or without incomplete Freund’s adjuvant	24 RRMS or SPMS	i.m. or i.d.	TCR peptides in saline (i.d.): injections on week 2,3,4,8,16,20 TCR peptides/IFA (i.m.) or IFA alone: injections 4,8,12, 16, 20	Phase 1/2	Three arm, randomized, partially blinded	24 w	Immunogenicity and safety	**Overall:** safe **Clinical:** no significant changes in EDSS, no significant differences in MRI activity between TCR responders/non responders **Immunological:** TCR peptide/IFA strong T cell response
(52) 2008	Trivalent TCR BV5S2, BV6S5, BV13S1 CDR2 peptides emulsified in IFA	14 RRMS, 10 SPMS 3 PPMS	i.m.	Monthly injections, 12 in total	Phase 1	Open label	54 w	Induction of TCR-specific T cells and response of PBMC	**Clinical:** 19 patients stable EDSS, 4 worse **Immunological:** development of IL-10 secreting TCR-peptide-specific T cells, increased expression of FoxP3 by Tregs and PBMC
NCT02057159 Started in 2017	Trivalent TCR emulsified in IFA	200 SPMS			Phase 2b	Randomized, double-blind, placebo-controlled, two arm parallel design		**Primary outcome**: cumulative number of new Gd-lesions up to 48 weeks **Secondary:** clinical relapses, EDSS score, immunological evaluations	unpublished
(53) 2010	Recombinant TCR ligand	11 RRMS 23 SPMS	i.v.	Doses of 2,6,20,60,200 or 100mg	Phase 1	Double-blind, placebo-controlled, dose-escalation	3mo	**Primary outcomes:** maximum tolerated dose, safety and tolerability **Secondary:** evaluation of pharmacokinetics	**Safety:** Maximum tolerated dose was 60mg, doses >100mg caused hypotension and diarrhea. No evidence of disease activation, no worsening of MS **Clinical:** n.a. **Immunology:** no reduction in IL-6, MIP-1α,
(54) 2000	APL NBI-5788 derived from MBP_83–99_	142 RRMS	s.c.	5, 20 or 50mg weekly	Phase 2	Double-blind, randomized, placebo-controlled		**Primary outcome**: number of new Gd-enhancing lesions	**Safety:** trial suspended due to hypersensitivity reaction in 9% of the patients **Clinical:** 5mg dose reduced volume and number of Gd-lesions **Immunological:** Activation of a non-encephalitogenic autoimmune Th2 response
(13) 2000	APL ^1^CGP77116	8 RRMS	s.c.	50mg weekly, 1 patient 5 mg	Phase 2a	Open label, MRI- controlled		**Primary outcome**: change in mean number Gd-enhancing lesions **Secondary**: change in mean T2 white matter lesion load, change in EDSS, relapse rate, precursor frequency of MBP- or CGP77116-specifc T cells	**Safety:** trial halted due to *62%* increase in number of active lesions and disease exacerbations in two patients **Clinical:** n.a, **Immunological:** negative; “encephalitogenic” response in 3/8 patients
(55) 2000	Solubilized MBP_84–102_ complexed with MHC class II molecule DR2 (DRA/DRB1*15:01) AG284	33 SPMS	i.v.	0.6, 2.0, 6.0, 20.0, 60.0, 105.0, and 150.0 mg/kg body weight.	Phase 1	Placebo-controlled, double-masked, dose escalation		**Primary outcome**: safety and tolerability **Secondary:** comparison of pre- and post-treatment Gd-lesions, EDSS	**Safety:** safe and well tolerated. **Clinical:** negative, no effect on clinical or MRI activity **Immunological:** negative, no tolerization effect, no sustained conversion to MBP, MOG reactivity
(56) 1997	MBP_75-95_ MBP_82-98_ or MBP_86-95_	53 Chronic progressive MS	i.v., intra-thecal or s.c.	intrathecal:1 to 10mg iv: single or two injections of max. 500mg s.c.: increasing amounts of 1-100mg	Phase 1	Open-label	12mo	**Outcomes:** induction of antigen-specific tolerance, identification of suitable dose and route of administration	**Overall:** Only i.v. injection induced tolerance, no side effects **Clinical:** n.a. **Immunological:** MBP auto-ab undetectable for 3-4 mo, after second injection auto-ab undetectable after 1 y
(57) 2011 (NCT00468611)	Soluble MBP-derived peptide (MBP8298)	612 SPMS	i.v.	500mg once every 6 mo	Phase 3	Randomized, double-blind, placebo-controlled	2y	**Primary outcome:** time to progression by 1.0 EDSS point 6 mo later **Secondary:** mean change in EDSS, MRI changes, annual relapse rate, Quality of Life	**Safety:** overall safe **Clinical:** negative, no significant differences between treatment groups in both primary and secondary endpoint parameters **Immunological:** n.a.
(58) 2013	Skin patch loaded with MBP_85–99_, MOG_35–55_ and PLP_139–155_	30 RRMS	Transdermal	Weekly patch with 1mg or 10mg, then 1x per month for 11 months.	Phase 2	Placebo-controlled	12mo	**Primary outcome:** cumulative number of Gd-lesions in 1y **Secondary:** new T2, T1 lesions volume change from baseline to end of study, annual relapse rate, proportion of relapse-free patients, proportion of patients with 3 mo of confirmed disability worsening of EDSS at month 12	**Safety**: Safe and well tolerated **Clinical:** 1mg patch showed 66% reduction in cumulative number of Gd lesions, annual relapse rate lower compared to placebo **Immunology**: induction of IL-10 producing Tr1 cells
(24) 2015	ATX-MS-1467 (four MBP-derived peptides)	6 SPMS	i.d.	6 injections at 7 to 14 day intervals (starting from 25µg, 50, 100,400 and 800µg + second injection of 800µg)	Phase 1	Open-label dose escalation study		**Primary outcome**: safety assessment	**Safety:** safe and well tolerated **Clinical**: n.a. **Immunological**: trend towards higher IL-10 expression
(59) 2018	Apitopes ATX-MS-146 (consisting of four MBP-derived peptides)	37 RRMS	i.d.	Dose titration from 50µg on day 1, 200µg day 15, 800µg on day 29, then biweekly administration of 800µg for 16 weeks	Phase 2a	Open label single arm, baseline-controlled	36 w	**Primary outcome**: change in number of Gd-lesions on treatment vs. baseline. **Secondary:** number of new T2, ARR at week 20, time to first relapse, EDSS change, MSFC **Safety:** AEs, injection site reaction, Safety and tolerability number of Gd at month 3-5 compared to baseline	**Safety:** injection site reactions **Clinical:** number of Gd-lesions significantly reduced, changes in EDSS, MSFC not significant **Immunological:** n.a.
(60, 61) 2016	MBP peptides co-encapsulated in CD206-targeted liposomes	16 RRMS 4 SPMS	s.c.	6x weekly applications, doses ascending from 50µg – 900µg. Total dose 2.675mg	Phase 1/2a	Open label, dose-escalating,	18w	**Primary endpoint**: safety, determined by frequency and severity of AEs and SAEs **Secondary:** number of relapses, EDSS at end of trial, number of Gd lesions, and total number of T2 lesions, concentration of pro- and anti-inflammatory cytokines	**Safety:** positive: safe and tolerable. **Clinical:** negative. EDSS, T2-weighted and Gd-lesions unchanged **Immunological**: monocyte chemoattractant protein-1, macrophages inflammatory protein 1b and IL-7 decreased, TNF-α increased
**Cell-based approaches**										
**Ref. & year**	**Substance**	**Number patients/MS type**	**R. of admin.**	**Dose/Frequency**	**Study phase**	**Study design**	**Trial duration**	**Outcome parameters**	**Results**
(62) 1995 *	Irradiated T cells reactive to myelin basic	5 RRMS 3 PMS	s.c.	3 injections	Phase 1	Open label	2-3y	Changes in exacerbation rate, EDSS and brain MRI lesions	**Clinical:** lesions and relapses worsened in 3 cases reappearance of MBP-reactive T cells, MRI: treatment group 8% increase in brain lesion size, 39.5% in control group
(63) 2000	Bovine myelin-reactive irradiated T cells	4 SPMS	s.c.	3-monthly over 24 months	Phase 1	Open label	30-39mo	Immunological und clinical response	**Clinical:** 2 patients stable EDSS, one improved EDDS, one advancing EDSS **Immunological:** decrease of myelin-reactive T cells, IL-2 and IFN-y secreting T cells
(64) 2002	Irradiated autologous MBP-reactive T-cells	28 RRMS 26 SPMS	s.c.	3 injections at 2 months intervals	Phase 1	Open label	24mo	Time to onset of confirmed progression of disability, EDSS, rate of relapse and MRI lesion, safety assessment **Others:** immune response as measured by T-cell activity in CSF	**Overall:** depletion of MBP-reactive T cells correlated with a 40% reduction in relapse rate **Clinical:** minimal EDSS reduction in RRMS, slight increase in EDSS in SPMS patients. Accelerated disease progression after 12 mo. MRI: slight reduction in lesion activity **Immunological:** reappearance of MBP-specific T cells after 12 months in 10-12% of patients
(65) 2003	CSF-derived autologous attenuated CD4+ T cells	4 RRMS 1 CPMS	s.c.	3 times 10 Mio. Cells, interval of 2 months	Phase 1	Open-label	15mo	Safety, feasibility and immune effects	**Safety:** well tolerated, no toxicity or AEs **Clinical**: stable patients **Immunological**: anti-ergotypic response in all patients, anti-MBP, MOG or PLP reactivities low or reduced
(66) 2000	Mixture of attenuated myelin reactive T cells	9 RRMS 7 SPMS	s.c.	Injections at week 0,4,12,20 In 3 different doses	Phase 1	Open-label dose-escalation	52 w	Clinical parameter (EDSS, MRI, relapses), levels of myelin reactive T cells.	**Safety:** AE mild to moderate **Clinical:** trend in EDSS improvement, stable MRI **Immunological**: medium dose most effective in reducing myelin-reactive T cells, whereas high dose led to increase
(67) 2012	Irradiation-attenuated myelin-reactive T cells by	26 relapsing-progressive MS	s.c.	4 injections of 10-30x10^6^ T cells on day 1,30,90, 180	Phase 1/2	Randomized double-blind, controlled	1y	Safety and efficacy	**Overall:** safe and feasible **Clinical:** decrease of EDSS and 10-Meter walking time, 94% remained relapse free/placebo group 43%) No significant change in MRI parameters
NCT01684761	Myelin-reactive T-cells (Tcelna)	SPMS	s.c.	30-45x10^6^ T cells, 2 annual cycles of 5 doses (at week 0,4,8,12,24)	Phase 2	Double-blind, placebo-controlled	2y	**Primary outcome:** brain atrophy at 2y **Secondary:** disease progression at 2y	Unpublished
(15) 2013	Autologous peptide-coupled PBMCs	8 RRMS 2 SPMS	i.v.	Ten different doses in 10 patients: 1x10^3^, 1x10^5^, 1x10^7^, 1x10^8^ 5x10^8^, 1x10^9^, 1x10^9^ 2.5x10^9^ 3x10^9^	Phase 1	Open-label dose escalation baseline-to-treatment design	6mo	**Primary outcome:** safety and tolerability	**Safety results:** safe and well tolerated, **Clinical:** n.a. **Immunological outcomes:** patients in higher dose group showed a decrease in antigen-specific T cells responses
(17) 2019	Tolerogenic dendritic cells	8 MS (SPMS, PPMS or RRMS) 4 NMOSD	i.v.	3 independent doses (cell doses ranging from 50x10^6^, 100x10^6^, 150x10^6^, 300x10^6^) administered every 2 weeks,	Phase 1b	Open-label, multiple ascending dose	24w	**Primary outcome**: safety and tolerability **Secondary:** clinical (relapses, disability), MRI, OCT, immunological response	**Safety:** safe and well tolerated **Clinical:** patients remained clinically stable, no new Gd-enhancing lesions **Immunological**: increased IL-10 production and frequency of Tr1 cells
(41) 2019	Autologous peptide-coupled RBCs	10 RRMS	i.v.	3 doses ranging from 1x10^10^ (2 patients), 1x10^11^ (3 patients), 3x10^11^ (5 patients) cells	Phase 1b	Open-label baseline-to-treatment design	6mo	**Primary:** safety and feasibility	unpublished
NCT02618902 NCT02903537 NCT02283671	Dendritic cells pulsed with myelin-derived peptides	MS	i.v. or i.d. or intra-nodal		Phase 1	Dose-escalating		**Primary:** safety and feasibility **Secondary:** changes in EDSS	ongoing

*older studies not matching current reporting standards.

#, number; ab, antibody; APL, altered peptide ligand; CDR2, complementarity determining region 2; CPMS, chronic progressive MS; EDSS, Expanded disability status scale; Gd, Gadolinium; IL, interleukin; i.d., intradermal i.m., intramuscular; i.v., intravenous; mo, months; MRI, magnet resonance imaging; n.a., not applicable; NMOSD, Neuromyelitis optica spectrum disorder; OCT, optical coherence tomography; PBMC, peripheral blood mononuclear cells; PPMS, primary progressive MS; Route of admin, route of administration; ref., reference; RBC, red blood cells; RRMS, relapsing-remitting; s.c., subcutaneous; SPMS, secondary progressive MS; TCR, T cell receptor; we, weeks; y, year.

A pioneering approach aimed at tolerization through oral administration of whole bovine myelin. A randomized, placebo-controlled clinical trial in early relapsing-remitting MS (RRMS) patients failed the primary endpoint of reducing clinical disease activity, including the number of relapses and disability progression, despite promising data on antigen-specific T cells (47). Both gender and HLA haplotypes were unequally distributed between the treatment groups, limiting the interpretation of results and already pointing at the importance of patient stratification in tolerization trials (68). A double-blind, phase 3 clinical trial of a single dose of bovine myelin in 515 MS patients failed to show a reduction in relapses, however, an extraordinarily strong placebo effect was observed, which might have influenced the result (69). The formulation and the dose might have been additional factors leading to the failure of the approach, since the human equivalent dose was lower than the effective dose in mice.

Another RoA was explored by intravenous administration of a soluble MBP82-98 peptide, which was well tolerated and showed a beneficial effect on disease progression in HLA DR2+ secondary-progressive MS (SPMS) patients (56, 70). An increase of regulatory T cells up until 6 months post treatment was shown, and interestingly a reversal of the T cell anergic state was seen in the high dose group (71). However, a phase 3 trial failed with no significant benefit over placebo with respect to reducing disease progression (57). The results of the trial emphasize the importance of choosing the optimal disease stage for tolerization approaches. At later stages like SPMS, it is probably not only challenging to curb a long-lasting autoimmune response, but also much more difficult to prove an effect in clinical trials.

An important lesson came from a clinical trial with an altered peptide ligand (APL) of the immunodominant MBP83-99 peptide, which led to induction of new disease activity in RRMS patients. APLs are generated through modification of amino acids in TCR contact positions, which can block or alter T cell responses through serving as partial agonist and antagonist. Despite compelling evidence from pre-clinical studies (72) a phase 2a trial using MBP83-99-derived APL was halted due to induction of MS disease activity through stimulation of encephalitogenic MBP83-99 reactive T cells (13). Thus far, this is the only evidence in humans that a MBP-specific immune response can trigger inflammatory lesions and relapses in MS patients. Further, the study demonstrated the importance of thorough safety monitoring by clinical and imaging measures in early phase trials. Whether the unusually low number of DR15+ MS patients contributed to the outcome is currently not clear, but possible. Hypersensitivity reactions led to discontinuation of a second and larger phase 2 trial with the same APL given at three doses, although there were signs of a beneficial effect on the number of contrast-enhancing lesions (54, 73).

A mix of four processing-independent MBP peptides (ATX-MS-1467) for subcutaneous or intradermal application, was safe and well tolerated in a phase 1 and successful in a phase 2 trial in RRMS patients showing a significant reduction of new and total contrast-enhancing lesions (24, 59). However, the trial also demonstrated the limitations of an antigen-specific therapy in patients with very high disease activity. Further trials are warranted to confirm the beneficial effect of the approach and to assess whether it might lead to a long-lasting therapeutic effect, that is persisting immune tolerance, or may need continuous administration of the AST.

Different from the above modalities, BHT-3009 builds on ectopic expression of a myelin protein *via* intramuscular injection of a plasmid encoding full length MBP, which leads to muscle cells expressing sustained low levels of MBP. BHT-3009 demonstrated promising effects on radiological disease activity in active MS patients in a phase 1/2 study, a reduction of MBP-specific CD4+ T cells with a Th1 phenotype in peripheral blood and a decrease of myelin-specific auto-antibody titers in CSF (45). A subsequent phase 2 trial did not meet the primary endpoint in reducing the number of new contrast-enhancing lesions (46). Overall, the approach remains promising and is currently followed in type 1 diabetes and neuromyelitis optica (NMO) (https://tolerion.bio/pipeline/).

Inducing immune tolerance to peptides of different myelin proteins simultaneously, including MBP, MOG and PLP promises to increase the efficacy of the treatment. Transdermal administration of three myelin peptides (MBP85-99, MOG35-55 and PLP139-155) *via* skin patches was one of the first ASTs in MS to demonstrate efficacy in reducing clinical- and MRI disease activity in RRMS patients (58). Peptide application led to local activation of Langerhans cells, reduced myelin peptide-specific T cell responses and increases of IL-10-secreting T cells (74).

Our group employed chemical coupling of seven myelin peptides from MOG, MBP and PLP (MOG1-20, MOG35-55, MBP13-32, MBP83-99, MBP111-129, MBP146-170, PLP139-154), (see above) to autologous peripheral blood mononuclear cells (PBMC) (14, 15). The approach targets the highest number of antigens based on the above considerations (see **Table 1**, **Box 1**) and was safe and well tolerated in a phase 1b study in RRMS and SPMS patients with T cell reactivity against at least one of the myelin peptides (15). Mechanistic studies including immunophenotyping of immune cell populations, cytokine responses and both anti-myelin and -non-myelin autoantibodies did not show any signs of induction of autoreactivity (15, 75). In patients receiving high doses a reduction of myelin peptide-specific T cell responses was observed after treatment. To improve the tolerization regimen by targeting both liver and spleen as important tolerogenic organs, we switched to autologous red blood cells (RBC) as carrier cells and assessed the safety and feasibility of autologous myelin peptide-coupled RBCs in a phase 1b trial (41). Following these promising results, a phase 2 study is currently in preparation.

Besides peptide-coupled fixed carrier cells, several groups explore antigen-loaded dendritic cells (DCs) with a good safety and tolerability profile in a phase 1b study in MS (seven myelin peptides) and NMO (aquaporin-4 peptides) patients (17). Increased production of IL-10 and of Tr1 cell numbers were observed. Currently, three different open label phase 1 studies are conducted to evaluate myelin peptide-pulsed DCs in different immature/tolerogenic states and given by different routes (intravenous, intradermally and intranodally; NCT02618902, NCT02903537, NCT02283671).

An alternative AST strategy aims to induce an immune response against important effector mechanisms of the autoreactive immune system, for example the autoreactive T cell clone or its TCRs. The appeal is that induction versus abrogation of immune responses might be easier to achieve. There have been various promising studies with T cell- or T cell receptor (TCR) vaccination, which were well tolerated and led to a reduction of myelin-reactive and IL-2- or IFN-γ secreting T cells (62–67, 76–79). Renewed disease progression 12 months after the last vaccination indicated that refresher injections are needed (65), as did reappearing MBP-specific TCC, which could be eliminated by additional vaccination (78).

MBP-specific T cells from MS patients frequently express specific TCR variable chains Vβ5.2 and Vβ6.1 (80, 81). Several trials in MS patients using intradermal or intramuscular injections of synthetic TCR Vβ5.2 and/or and Vβ6.1 peptides reported clinical improvements and reduced frequency of MBP-specific T cells and the induction of TCR peptide-specific T cells (48–50). The administration of a trivalent TCR vaccine induced TCR-peptide specific T cells secreting IL-10 and increased the expression of FoxP3 in Tregs, which was paralleled by a reduction in MOG145-160 specific T cells, suggesting the induction of a regulatory network by the vaccine (51, 52) (NCT02057159).

## Challenges and Prospects in the Clinical Development of Antigen-Specific Therapies

**Figure 2:**
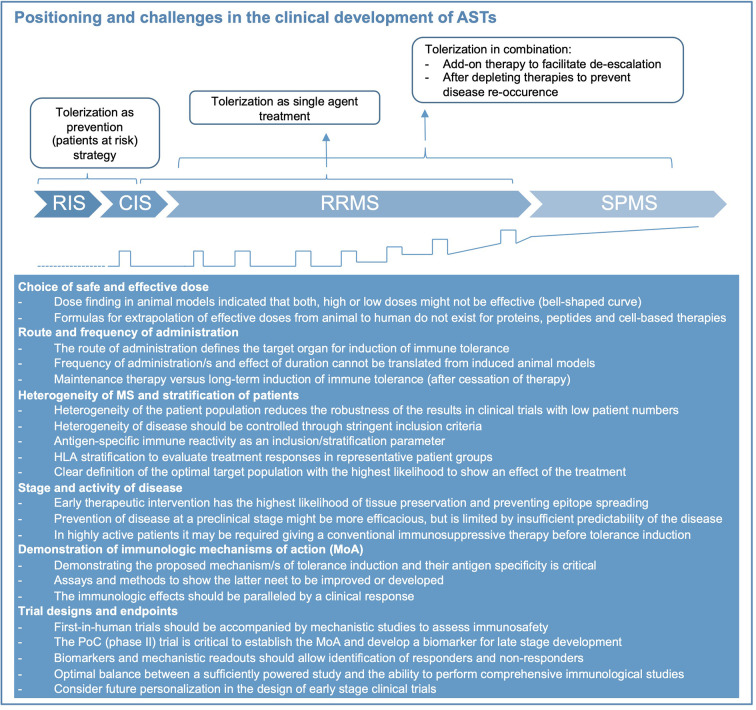
Positioning of immune tolerance in disease stages of MS and key challenges for treatment development. Considerations how tolerization appears most meaningful during the different disease stages of MS (represented also graphically at bottom of figure, relapses indicated by open squares). Abbreviations: CIS, clinically isolated syndrome; RIS, radiologically isolated syndrome; RRMS, relapsing-remitting MS; SPMS, secondary-progressive MS. During RIS and CIS, as well as following highly active immunomodulatory therapy tolerization aims at preventing further evolution or re-occurrence of disease. As single agent treatment during RRMS, tolerization aims at replacing currently approved therapies. Its role in SPMS and primary progressive (PP) MS (not shown) is speculative at present.

Several tolerization strategies in MS were safe and feasible in phase 1 studies but the consecutive phase 2 and -3 trials remained unsatisfying. It has become clear that the clinical development of antigen-specific therapies poses distinct challenges that need to be tackled along the clinical trial program. Several aspects of the disease like inflammatory activity, progression or disease stage, patient selection, characteristics of the tolerizing product such as RoA, dose and interval of administration, concomitant therapies need to be considered, and each step is difficult (**Figure 2**). Developing an optimal trial design therefore remains very demanding.

Certainly, an important challenge in the early proof-of-concept studies is the heterogeneity of the disease with regard to genetic background, main pathomechanisms, and clinical aspects including disease form, course and response to treatments. The HLA DR-15 haplotype is by far the most important susceptibility gene and key in shaping antigen-specific immune responses. Consequently, the individual HLA background might contribute to heterogeneity of antigen-specific immune responses and influence the efficacy of ASTs. The individual’s HLA type therefore needs to be considered as already demonstrated in the early oral tolerance trial (47), and particularly during early clinical development, one should assure that a representative population of patients is included.

Ideally, the tolerization approach should be administered early in the disease and block epitope spreading (**Figure 2**). Although several studies have reported promising immunological effects of tolerization in SPMS patients, they failed to show efficacy on clinical and imaging measures of inflammatory disease activity and disability progression. Thus, both disease duration and stage should be considered, and ideally biomarkers including antigen-specific immune responses should be used for stratification of patients. The intensity and extent of inflammatory disease activity, which is often higher during the early stage of the disease is another important factor. Since it may take some time until tolerization becomes effective, it may be necessary to start the tolerizing therapy in combination with an effective anti-inflammatory treatment to decrease disease activity and provide the optimal environment for induction of tolerance. Future trials should consider this aspect particularly in highly active patients and explore the optimal duration for a combination therapy before switching to monotherapy with the tolerizing agent (conceptual considerations shown in **Figure 2**). For such sequential application or combined use of tolerizing therapies the specific immunologic effects of the conventional immune therapy, its durability and how this might interfere with the main mechanisms of immune tolerance induced by the AST are important aspects to keep in mind. Immune therapies leading to a broad reduction or even long-term depletion of several or single lymphocyte subsets might also impede the generation of regulatory cell populations and thus dampen the tolerizing effects. More specific inhibition of immune cell activation/proliferation or trafficking of autoreactive lymphocytes to the CNS is less likely to interfere with the induction of immune tolerance and in case of the latter might even act synergistically by providing better exposure of autoreactive T and B cells to the tolerizing agent (11). The choice of a combination therapy will also depend on the timing of the treatment, i.e. whether the AST is applied early in the disease (for example as first-line therapy) or as part of a de-escalation strategy where the conventional immune therapy is already established.

Establishing the dose and frequency of administration of an AST are important aspects during clinical development. Prior studies have shown that the antigen dose might influence the mechanism of immune tolerance induced by the treatment (82). Extrapolation of an effective dose from rodents to humans is difficult, since, different from small molecules, accepted formulas do not exist for cell-based therapies or other novel strategies (e.g. nanoparticle-based approaches, DNA vaccination, and others). In addition, almost all animal models are induced and have a monophasic disease course, or the AST shows long-lasting, sometimes life-long, efficacy even after a single treatment (21), thus providing little guidance with respect to frequency of administration in patients. Consequently, there is a strong need for biomarkers that can be employed for dose finding and assessing the duration of the tolerization effect in AST trials.

As mentioned above the RoA of the target antigen is critical. It influences cell type and organ that take up the tolerizing peptide, nanoparticle or cell product and affects the type of immune response that the respective tolerizing approach produces. Oral application has long been considered ideal for the induction of tolerance due to the important role of the mucosal immune system, which assures tolerance to food antigens. However, clinical studies of oral tolerance did not yet show efficacy in MS, which may be related to the formulation, dose and choice of antigen. Subcutaneous (s.c.) administration is usually associated with immune activation (APL trial) as opposed to intravenous (i.v.) application (iv MBP 83-99), intradermal (i.d.) and transdermal (t.d.) application of peptides (59). Hypersensitivity reactions were an important issue in a phase 2 trial of subcutaneous administration of an APL (13, 54). The spleen is important for degradation of aged cells, and particularly the liver plays a role in immune tolerance to blood borne antigens (83). Therefore, i.v. application of antigens, peptide-coupled cells or nanoparticles have been considered most effective in targeting these organs and to be safe although hypersensitivity reactions have been observed in animal models (84).

Phase 1 testing of ASTs need to establish safety and tolerability and exclude proinflammatory activation of immune responses to the target antigens. Finding the right starting dose is critical for gene and cell-based approaches, but generally for ASTs (see above). The efficacy outcomes for early phase 2 clinical trials in MS are well established and mainly use MRI as a surrogate for inflammatory disease activity (85). Documenting efficacy on the surrogate outcome (MRI) should be accompanied by mechanistic studies, which support the putative MoA of the AST, and may identify subgroups of patients with strong or poor responsiveness and the optimal dose range (**Figure 2**). As outlined above, there is a need for a consensus on suitable MoA-oriented outcome parameters for tolerizing therapies. Approaching this goal, requires coordination and collaboration between research groups, which could build on ongoing initiatives like the Immune Tolerance Network in the US and dedicated scientific networks in Europe (86, 87), ideally with further involvement of competent authorities. Current highly active therapies reduce inflammatory MRI activity by 90% or more, which is unlikely to be improved by ASTs. However, since tolerance induction would be a completely new treatment modality and is expected to be superior with respect to safety and tolerability over short and particularly longer treatment courses, these aspects should be built into the clinical development strategy. Experiences in the past have shown that following the path of clinical testing that is well established for small molecules and biologics may not be ideal for ASTs. The current treatment landscape of MS offers several approved therapies for patients with high disease activity, but there is a lack of therapies that are safe, do not pose problems for women, who wish to get pregnant, and do not increase the risk for infections or damage organs. These considerations are particularly relevant for the increasing number of patients with low disease activity and those who are in the very early stages of the disease. Induction of immune tolerance potentially fills an unmet medical need for therapies that provide an ideal balance between efficacy and safety (**Figure 2**). Furthermore, a group of patients, which is currently not treated, are patients with radiological evidence for MS prior to any clinical symptom, i.e. radiologically isolated syndrome (RIS). RIS patients would greatly benefit from a therapy that does not lead to unspecific immunosuppression. Thus, tolerance-inducing therapies may fit best for patients at early or preclinical stages of the disease or as a sequential therapy after induction with highly effective immunomodulatory treatments in patients with high disease activity (**Figure 2**). Depending on how the identification of genetic risk profiles and biomarkers evolves, it can even be envisioned that that tolerance induction may be used prophylactically to prevent the onset of MS in the future.

## Future Directions to Improve Treatment Development

Induction of antigen-specific immune tolerance is an attractive treatment goal for MS and other autoimmune diseases, and different strategies are in pre-clinical and clinical stages. We outline key points that should be considered to improve the development of tolerizing therapies and to find the best way how to fit them into our current treatment algorithms. These include validation of biomarkers to measure induction of immune tolerance, definition of relevant MoA of each strategy and documentation of long-term reduction in antigen-specific immune responses parallel to effects on clinical outcome parameters. Clinical trial designs need to be improved and tailored to the specific challenges that AST pose. Consensus criteria for AST trials should be developed. If the above challenges can be mastered, the successful application of AST in any autoimmune disease would represent a major breakthrough in medicine and enter a new treatment era that aims at treating autoimmunity with high specificity and minimal side effects or even preventing its development.

## Author Contributions

All authors declare that they have substantially participated in the preparation and writing of the manuscript and have taken due care regarding their contribution to ensure the integrity of the work. All authors contributed to the article and approved the submitted version.

## Funding

HH-K, AL, RM, and MS are supported by the Clinical Research Priority Program of the University of Zurich (Precision^MS^) and the Wyss Zurich. Swiss National Fund Project#310030_197652 (A Lutterotti).

## Conflict of Interest

AL received financial compensation and/or travel support for lectures and advice from Biogen, Merck, Novartis, Teva, Genzyme, Bayer, Celgene and he is a co-founder and co-owner of Cellerys, a company which pursues antigen-specific tolerization. He is co-inventor on a patent held by the University of Zurich on the use of peptide-coupled cells for treatment of MS. HH-K received compensation for advice, lecturing or travel support from Biogen, Genzyme, Merck, Novartis, and Teva. MS is a co-founder and co-owner of Cellerys, which pursues antigen-specific tolerization. Together with RM, she is an inventor on a Univ. Zurich-held patent of target antigens GDP L-fucose synthase and RAS guanyl-releasing protein 2 for multiple sclerosis. RM received unrestricted grant support from Biogen and Novartis, and compensation for advice or lecturing by Biogen, Novartis, Sanofi Genzyme, Merck, Hoffmann La Roche, Teva, Neuway, CellProtect, and Third Rock Ventures. He is a co-founder and co-owner of Cellerys, which pursues antigen-specific tolerization. Together with MS, he is an inventor on a Univ. Zurich-held patent of target antigens GDP L-fucose synthase and RAS guanyl-releasing protein 2 for multiple sclerosis.
