# Supplementary material for: Antigen-Specific Immune Tolerance in Multiple Sclerosis—Promising Approaches and How to Bring Them to Patients
Source: Front Immunol. 2021 Mar 22;12:640935. doi: 10.3389/fimmu.2021.640935 (PMC8019937; doi:10.3389/fimmu.2021.640935)
Supplement: Supplementary file 1 [file DataSheet_1.docx]

Supplementary Material

# Supplementary Data

**References for Table 1**

S1. M. Vergelli *et al.*, T cell response to myelin basic protein in the context of the multiple sclerosis-associated HLA-DR15 haplotype: Peptide binding, immunodominance and effector functions of T cells, *J Neuroimmunol* **77**, 195-203 (1997).

S2. R. Martin, H. F. McFarland, D. E. McFarlin, Immunological aspects of demyelinating diseases, *Annu Rev Immunol* **10**, 153-187 (1992).

S3. H. Acha-Orbea *et al.*, Limited heterogeneity of T cell receptors from lymphocytes mediating autoimmune encephalomyelitis allows specific immune intervention, *Cell* **54**, 263-273 (1988).

S4. R. B. Fritz, D. E. McFarlin, Encephalitogenic epitopes of myelin basic protein, *Chem Immunol* **46**, 101-125 (1989).

S5. R. B. Fritz, M. J. Skeen, C. H. Chou, S. S. Zamvil, Localization of an encephalitogenic epitope for the SJL mouse in the N-terminal region of myelin basic protein, *J Neuroimmunol* **26**, 239-243 (1990).

S6. J. L. Urban *et al.*, Restricted use of T cell receptor V genes in murine autoimmune encephalomyelitis raises possibilities for antibody therapy, *Cell* **54**, 577-592 (1988).

S7. S. S. Zamvil, L. Steinman, The T lymphocyte in experimental allergic encephalomyelitis, *Annu Rev Immunol* **8**, 579-621 (1990).

S8. B. Bielekova *et al.*, Expansion and functional relevance of high-avidity myelin-specific CD4+ T cells in multiple sclerosis, *J Immunol* **172**, 3893-3904 (2004).

S9. H. B. Streeter, R. Rigden, K. F. Martin, N. J. Scolding, D. C. Wraith, Preclinical development and first-in-human study of ATX-MS-1467 for immunotherapy of MS, *Neurol-Neuroimmunol* **2**, (2015).

S10. J. Chataway *et al.*, Effects of ATX-MS-1467 immunotherapy over 16 weeks in relapsing multiple sclerosis, *Neurology* **90**, E955-504 (2018).

S11. E. S. Huseby *et al.*, A pathogenic role for myelin-specific CD8(+) T cells in a model for multiple sclerosis, *J Exp Med* **194**, 669-676 (2001).

S12. Q. Ji, L. Castelli, J. M. Goverman, MHC class I-restricted myelin epitopes are cross-presented by Tip-DCs that promote determinant spreading to CD8(+) T cells, *Nat Immunol* **14**, 254-261 (2013).

S13. R. Martin *et al.*, A Myelin Basic-Protein Peptide Is Recognized by Cytotoxic T-Cells in the Context of 4 Hla-Dr Types Associated with Multiple-Sclerosis, *J Exp Med* **173**, 19-24 (1991).

S14. K. Ota *et al.*, T-cell recognition of an immunodominant myelin basic protein epitope in multiple sclerosis, *Nature* **346**, 183-187 (1990).

S15. R. Martin *et al.*, Fine specificity and HLA restriction of myelin basic protein-specific cytotoxic T cell lines from multiple sclerosis patients and healthy individuals, *J Immunol* **145**, 540-548 (1990).

S16. M. Pette *et al.*, Myelin basic protein-specific T lymphocyte lines from MS patients and healthy individuals, *Neurology* **40**, 1770-1776 (1990).

S17. L. S. Madsen *et al.*, A humanized model for multiple sclerosis using HLA-DR2 and a human T-cell receptor, *Nat Genet* **23**, 343-347 (1999).

S18. B. Bielekova *et al.*, Encephalitogenic potential of the myelin basic protein peptide (amino acids 83-99) in multiple sclerosis: Results of a phase II clinical trial with an altered peptide ligand (vol 6, pg 1167, 2000), *Nat Med* **6**, 1412-1412 (2000).

S19. J. A. Quandt *et al.*, Myelin Basic Protein-Specific TCR/HLA-DRB5*01:01 Transgenic Mice Support the Etiologic Role of DRB5*01:01 in Multiple Sclerosis, *Journal of Immunology* **189**, 2897-2908 (2012).

S20. M. Sospedra, R. Martin, Immunology of multiple sclerosis, *Annu Rev Immunol* **23**, 683-747 (2005).

S21. P. A. Muraro *et al.*, Immunodominance of a low-affinity major histocompatibility complex-binding myelin basic protein epitope (residues 111-129) in HLA-DR4 (B1*0401) subjects is associated with a restricted T cell receptor repertoire, *J Clin Invest* **100**, 339-349 (1997).

S22. J. A. Quandt *et al.*, Unique clinical and pathological features in HLA-DRB1*0401-restricted MBP 111-129-specific humanized TCR transgenic mice, *J Exp Med* **200**, 223-234 (2004).

S23. D. Sun, H. Wekerle, Ia-restricted encephalitogenic T lymphocytes mediating EAE lyse autoantigen-presenting astrocytes, *Nature* **320**, 70-72 (1986).

S24. C. Tal, P. K. Olitsky, Quantitative Studies on Proteolipide as Incitant of Disseminated Encephalomyelitis in Mice, *Science* **116**, 420-421 (1952).

S25. J. M. Greer, M. P. Pender, Myelin proteolipid protein: An effective autoantigen and target of autoimmunity in multiple sclerosis, *J Autoimmun* **31**, 281-287 (2008).

S26. S. Markovicplese *et al.*, T-Cell Recognition of Immunodominant and Cryptic Proteolipid Protein Epitopes in Humans, *J Immunol* **155**, 982-992 (1995).

S27. J. L. Trotter *et al.*, T cell recognition of myelin proteolipid protein and myelin proteolipid protein peptides in the peripheral blood of multiple sclerosis and control subjects, *J Neuroimmunol* **84**, 172-178 (1998).

S28. M. A. Friese *et al.*, Opposing effects of HLA class I molecules in tuning autoreactive CD8(+) T cells in multiple sclerosis, *Nat Med* **14**, 1227-1235 (2008).

S29. C. M. Pelfrey, J. L. Trotter, L. R. Tranquill, H. F. Mcfarland, Identification of a Novel T-Cell Epitope of Human Proteolipid Protein (Residues 40-60) Recognized by Proliferative and Cytolytic Cd4+ T-Cells from Multiple-Sclerosis Patients, *J Neuroimmunol* **46**, 33-42 (1993).

S30. C. M. Pelfrey, L. R. Tranquill, A. B. Vogt, H. F. McFarland, T cell response to two immunodominant proteolipid protein (PLP) peptides in multiple sclerosis patients and healthy controls, *Mult Scler* **1**, 270-278 (1996).

S31. K. Kawamura *et al.*, Hla-DR2-restricted responses to proteolipid protein 95-116 peptide cause autoimmune encephalitis in transgenic mice, *J Clin Invest* **105**, 977-984 (2000).

S32. T. Kondo *et al.*, TCR repertoire to proteolipid protein (PLP) in multiple sclerosis (MS): homologies between PLP-specific T cells and MS-associated T cells in TCR junctional sequences, *Int Immunol* **8**, 123-130 (1996).

S33. C. M. Pelfrey, J. L. Trotter, L. R. Tranquill, H. F. McFarland, Identification of a novel T cell epitope of human proteolipid protein (residues 40-60) recognized by proliferative and cytolytic CD4+ T cells from multiple sclerosis patients, *J Neuroimmunol* **46**, 33-42 (1993).

S34. C. M. Pelfrey, J. L. Trotter, L. R. Tranquill, H. F. McFarland, Identification of a second T cell epitope of human proteolipid protein (residues 89-106) recognized by proliferative and cytolytic CD4+ T cells from multiple sclerosis patients, *J Neuroimmunol* **53**, 153-161 (1994).

S35. V. K. Tuohy, Z. J. Lu, R. A. Sobel, R. A. Laursen, M. B. Lees, A synthetic peptide from myelin proteolipid protein induces experimental allergic encephalomyelitis, *J Immunol* **141**, 1126-1130 (1988).

S36. H. Waldner, M. J. Whitters, R. A. Sobel, M. Collins, V. K. Kuchroo, Fulminant spontaneous autoimmunity of the central nervous system in mice transgenic for the myelin proteolipid protein-specific T cell receptor, *Proc Natl Acad Sci U S A* **97**, 3412-3417 (2000).

S37. M. P. Pender *et al.*, Surges of increased T cell reactivity to an encephalitogenic region of myelin proteolipid protein occur more often in patients with multiple sclerosis than in healthy subjects, *J Immunol* **165**, 5322-5331 (2000).

S38. W. Zhao, K. W. Wegmann, J. L. Trotter, K. Ueno, W. F. Hickey, Identification of an N-terminally acetylated encephalitogenic epitope in myelin proteolipid apoprotein for the Lewis rat, *J Immunol* **153**, 901-909 (1994).

S39. C. C. Bernard *et al.*, Myelin oligodendrocyte glycoprotein: a novel candidate autoantigen in multiple sclerosis, *J Mol Med (Berl)* **75**, 77-88 (1997).

S40. E. Wallstrom, R. Weissert, J. Lorentzen, T. Olsson, Major histocompatibility complex haplotype RT1av1 is associated with relapsing/remitting experimental autoimmune encephalomyelitis, *Transplant Proc* **29**, 1686-1689 (1997).

S41. M. Adelmann *et al.*, The N-terminal domain of the myelin oligodendrocyte glycoprotein (MOG) induces acute demyelinating experimental autoimmune encephalomyelitis in the Lewis rat, *J Neuroimmunol* **63**, 17-27 (1995).

S42. T. G. Johns *et al.*, Myelin oligodendrocyte glycoprotein induces a demyelinating encephalomyelitis resembling multiple sclerosis, *J Immunol* **154**, 5536-5541 (1995).

S43. M. Bronge *et al.*, Myelin oligodendrocyte glycoprotein revisited-sensitive detection of MOG-specific T-cells in multiple sclerosis, *J Autoimmun* **102**, 38-49 (2019).

S44. C. P. Genain *et al.*, Antibody facilitation of multiple sclerosis-like lesions in a nonhuman primate, *J Clin Invest* **96**, 2966-2974 (1995).

S45. C. Linington *et al.*, T cells specific for the myelin oligodendrocyte glycoprotein mediate an unusual autoimmune inflammatory response in the central nervous system, *Eur J Immunol* **23**, 1364-1372 (1993).

S46. N. Kerlero de Rosbo *et al.*, Predominance of the autoimmune response to myelin oligodendrocyte glycoprotein (MOG) in multiple sclerosis: reactivity to the extracellular domain of MOG is directed against three main regions, *Eur J Immunol* **27**, 3059-3069 (1997).

S47. A. Iglesias, J. Bauer, T. Litzenburger, A. Schubart, C. Linington, T- and B-cell responses to myelin oligodendrocyte glycoprotein in experimental autoimmune encephalomyelitis and multiple sclerosis, *Glia* **36**, 220-234 (2001).

S48. E. Wallstrom *et al.*, Increased reactivity to myelin oligodendrocyte glycoprotein peptides and epitope mapping in HLA DR2(15)+ multiple sclerosis, *Eur J Immunol* **28**, 3329-3335 (1998).

S49. N. K. de Rosbo, A. Ben-Nun, T-cell responses to myelin antigens in multiple sclerosis; relevance of the predominant autoimmune reactivity to myelin oligodendrocyte glycoprotein, *J Autoimmun* **11**, 287-299 (1998).

S50. I. Mendel, N. Kerlero de Rosbo, A. Ben-Nun, A myelin oligodendrocyte glycoprotein peptide induces typical chronic experimental autoimmune encephalomyelitis in H-2b mice: fine specificity and T cell receptor V beta expression of encephalitogenic T cells, *Eur J Immunol* **25**, 1951-1959 (1995).

S51. E. Bettelli *et al.*, Myelin oligodendrocyte glycoprotein-specific T cell receptor transgenic mice develop spontaneous autoimmune optic neuritis, *J Exp Med* **197**, 1073-1081 (2003).

S52. T. G. Forsthuber *et al.*, T cell epitopes of human myelin oligodendrocyte glycoprotein identified in HLA-DR4 (DRB1*0401) transgenic mice are encephalitogenic and are presented by human B cells, *J Immunol* **167**, 7119-7125 (2001).

S53. J. Klehmet *et al.*, T cell epitope spreading to myelin oligodendrocyte glycoprotein in HLA-DR4 transgenic mice during experimental autoimmune encephalomyelitis, *Clin Immunol* **111**, 53-60 (2004).

S54. R. B. Lindert *et al.*, Multiple sclerosis: B- and T-cell responses to the extracellular domain of the myelin oligodendrocyte glycoprotein, *Brain* **122 ( Pt 11)**, 2089-2100 (1999).

S55. B. Pollinger *et al.*, Spontaneous relapsing-remitting EAE in the SJL/J mouse: MOG-reactive transgenic T cells recruit endogenous MOG-specific B cells, *J Exp Med* **206**, 1303-1316 (2009).

S56. R. Weissert *et al.*, High immunogenicity of intracellular myelin oligodendrocyte glycoprotein epitopes, *J Immunol* **169**, 548-556 (2002).

S57. N. K. Koehler, C. P. Genain, B. Giesser, S. L. Hauser, The human T cell response to myelin oligodendrocyte glycoprotein: a multiple sclerosis family-based study, *J Immunol* **168**, 5920-5927 (2002).

S58. A. Shetty *et al.*, Immunodominant T-cell epitopes of MOG reside in its transmembrane and cytoplasmic domains in EAE, *Neurol Neuroimmunol Neuroinflamm* **1**, e22 (2014).

S59. M. Varrin-Doyer *et al.*, MOG transmembrane and cytoplasmic domains contain highly stimulatory T-cell epitopes in MS, *Neurol Neuroimmunol Neuroinflamm* **1**, e20 (2014).

S60. J. A. Maatta, M. S. Kaldman, S. Sakoda, A. A. Salmi, A. E. Hinkkanen, Encephalitogenicity of myelin-associated oligodendrocytic basic protein and 2 ',3 '-cyclic nucleotide 3 '-phosphodiesterase for BALB/c and SJL mice, *Immunology* **95**, 383-388 (1998).

S61. N. Kaushansky *et al.*, HLA-DQB1*0602 Determines Disease Susceptibility in a New "Humanized" Multiple Sclerosis Model in HLA-DR15 (DRB1*1501;DQB1*0602) Transgenic Mice, *J Immunol* **183**, 3531-3541 (2009).

S62. N. K. de Rosbo *et al.*, The myelin-associated oligodendrocytic basic protein region MOBP15-36 encompasses the immunodominant major encephalitogenic epitope(s) for SJL/J mice and predicted epitope(s) for multiple sclerosis-associated HLA-DRB1*1501, *Journal of Immunology* **173**, 1426-1435 (2004).

S63. A. Holz, B. Bielekova, R. Martin, M. B. A. Oldstone, Myelin-associated oligodendrocytic basic protein: Identification of an encephalitogenic epitope and association with multiple sclerosis, *Journal of Immunology* **164**, 1103-1109 (2000).

S64. J. F. Kaye *et al.*, The central nervous sytem-specific myelin oligodendrocytic basic protein (MOBP) is encephalitogenic and a potential target antigen in multiple sclerosis (MS), *J Neuroimmunol* **102**, 189-198 (2000).

S65. M. M. Morris-Downes *et al.*, Encephalitogenic and immunogenic potential of myelin-associated glycoprotein (MAG), oligodendrocyte-specific glycoprotein (OSP) and 2',3'-cyclic nucleotide 3'-phosphodiesterase (CNPase) in ABH and SJL mice, *J Neuroimmunol* **122**, 20-33 (2002).

S66. P. A. Muraro, M. Kalbus, G. Afshar, H. F. McFarland, R. Martin, T cell response to 2',3'-cyclic nucleotide 3'-phosphodiesterase (CNPase) in multiple sclerosis patients, *J Neuroimmunol* **130**, 233-242 (2002).

S67. M. J. Walsh, J. M. Murray, Dual implication of 2',3'-cyclic nucleotide 3' phosphodiesterase as major autoantigen and C3 complement-binding protein in the pathogenesis of multiple sclerosis, *J Clin Invest* **101**, 1923-1931 (1998).

S68. D. Lambracht-Washington *et al.*, Antigen specificity of clonally expanded and receptor edited cerebrospinal fluid B cells from patients with relapsing remitting MS, *J Neuroimmunol* **186**, 164-176 (2007).

S69. M. Soderstrom *et al.*, Autoimmune T cell repertoire in optic neuritis and multiple sclerosis: T cells recognising multiple myelin proteins are accumulated in cerebrospinal fluid, *J Neurol Neurosurg Psychiatry* **57**, 544-551 (1994).

S70. M. Andersson *et al.*, Multiple MAG peptides are recognized by circulating T and B lymphocytes in polyneuropathy and multiple sclerosis, *Eur J Neurol* **9**, 243-251 (2002).

S71. J. R. Moller, D. Johnson, R. O. Brady, W. W. Tourtellotte, R. H. Quarles, Antibodies to myelin-associated glycoprotein (MAG) in the cerebrospinal fluid of multiple sclerosis patients, *J Neuroimmunol* **22**, 55-61 (1989).

S72. D. Johnson *et al.*, Cell-mediated immunity to myelin-associated glycoprotein, proteolipid protein, and myelin basic protein in multiple sclerosis, *J Neuroimmunol* **13**, 99-108 (1986).

S73. S. Baig *et al.*, Multiple sclerosis: cells secreting antibodies against myelin-associated glycoprotein are present in cerebrospinal fluid, *Scand J Immunol* **33**, 73-79 (1991).

S74. H. Link *et al.*, Virus-reactive and autoreactive T cells are accumulated in cerebrospinal fluid in multiple sclerosis, *J Neuroimmunol* **38**, 63-73 (1992).

S75. A. Wajgt, M. Gorny, CSF antibodies to myelin basic protein and to myelin-associated glycoprotein in multiple sclerosis. Evidence of the intrathecal production of antibodies, *Acta Neurol Scand* **68**, 337-343 (1983).

S76. D. B. Stevens, K. Chen, R. S. Seitz, E. E. Sercarz, J. M. Bronstein, Oligodendrocyte-specific protein peptides induce experimental autoimmune encephalomyelitis in SJL/J mice, *J Immunol* **162**, 7501-7509 (1999).

S77. T. Vu, L. W. Myers, G. W. Ellison, F. Mendoza, J. M. Bronstein, T-cell responses to oligodendrocyte-specific protein in multiple sclerosis, *J Neurosci Res* **66**, 506-509 (2001).

S78. N. Kaushansky *et al.*, Epitope specificity of autoreactive T and B cells associated with experimental autoimmune encephalomyelitis and optic neuritis induced by oligodendrocyte-specific protein in SJL/J mice, *J Immunol* **177**, 7364-7376 (2006).

S79. J. J. Bajramovic *et al.*, Oligodendrocyte-specific protein is encephalitogenic in rhesus macaques and induces specific demyelination of the optic nerve, *Eur J Immunol* **38**, 1452-1464 (2008).

S80. N. Kaushansky, M. Eisenstein, J. H. Oved, A. Ben-Nun, Activation and control of pathogenic T cells in OSP/claudin-11-induced EAE in SJL/J mice are dominated by their focused recognition of a single epitopic residue (OSP58M), *Int Immunol* **20**, 1439-1449 (2008).

S81. M. Aslam *et al.*, The antibody response to oligodendrocyte specific protein in multiple sclerosis, *J Neuroimmunol* **221**, 81-86 (2010).

S82. R. Planas *et al.*, GDP-L-fucose synthase is a CD4(+) T cell-specific autoantigen in DRB3*02:02 patients with multiple sclerosis, *Sci Transl Med* **10**, (2018).

S83. I. Jelcic *et al.*, Memory B Cells Activate Brain-Homing, Autoreactive CD4(+) T Cells in Multiple Sclerosis, *Cell* **175**, 85-100 e123 (2018).

S84. K. Banki *et al.*, Oligodendrocyte-specific expression and autoantigenicity of transaldolase in multiple sclerosis, *J Exp Med* **180**, 1649-1663 (1994).

S85. E. Colombo *et al.*, Comparative analysis of antibody and cell-mediated autoimmunity to transaldolase and myelin basic protein in patients with multiple sclerosis, *J Clin Invest* **99**, 1238-1250 (1997).

S86. M. Esposito *et al.*, Human transaldolase and cross-reactive viral epitopes identified by autoantibodies of multiple sclerosis patients, *Journal of Immunology* **163**, 4027-4032 (1999).

S87. B. Niland, K. Banki, W. E. Biddison, A. Perl, CD8(+) T cell-mediated HLA-A*0201-restricted cytotoxicity to transaldolase peptide 168-176 in patients with multiple sclerosis, *Journal of Immunology* **175**, 8365-8378 (2005).

S88. J. M. Vannoort *et al.*, The Small Heat-Shock Protein Alpha-B-Crystallin as Candidate Autoantigen in Multiple-Sclerosis, *Nature* **375**, 798-801 (1995).

S89. A. C. van Sechel *et al.*, EBV-induced expression and HLA-DR-restricted presentation by human B cells of alpha B-crystallin, a candidate autoantigen in multiple sclerosis, *Journal of Immunology* **162**, 129-135 (1999).

S90. I. Saez-Torres *et al.*, Specific proliferation towards myelin antigens in patients with multiple sclerosis during a relapse, *Autoimmunity* **35**, 45-50 (2002).

S91. J. J. Bajramovic *et al.*, Presentation of alpha B-crystallin to T cells in active multiple sclerosis lesions: An early event following inflammatory demyelination, *Journal of Immunology* **164**, 4359-4366 (2000).

S92. S. S. Ousman *et al.*, Protective and therapeutic role for alphaB-crystallin in autoimmune demyelination, *Nature* **448**, 474-479 (2007).

S93. R. Verbeek, K. van der Mark, E. F. Wawrousek, A. C. Plomp, J. M. van Noort, Tolerization of an established alphaB-crystallin-reactive T-cell response by intravenous antigen, *Immunology* **121**, 416-426 (2007).

S94. J. M. van Noort, M. Bsibsi, P. J. Nacken, R. Verbeek, E. H. Venneker, Therapeutic Intervention in Multiple Sclerosis with Alpha B-Crystallin: A Randomized Controlled Phase IIa Trial, *PLoS One* **10**, e0143366 (2015).

S95. Y. K. Chou *et al.*, CD4 T-cell epitopes of human alpha B-crystallin, *Journal of Neuroscience Research* **75**, 516-523 (2004).

S96. E. K. Mathey *et al.*, Neurofascin as a novel target for autoantibody-mediated axonal injury, *J Exp Med* **204**, 2363-2372 (2007).

S97. M. Lindner, J. K. M. Ng, S. Hochmeister, E. Meinl, C. Linington, Neurofascin 186 specific autoantibodies induce axonal injury and exacerbate disease severity in experimental autoimmune encephalomyelitis, *Exp Neurol* **247**, 259-266 (2013).

S98. T. Derfuss, C. Linington, R. Hohlfeld, E. Meinl, Axo-glial antigens as targets in multiple sclerosis: implications for axonal and grey matter injury, *J Mol Med (Berl)* **88**, 753-761 (2010).

S99. T. Derfuss *et al.*, Contactin-2/TAG-1-directed autoimmunity is identified in multiple sclerosis patients and mediates gray matter pathology in animals, *P Natl Acad Sci USA* **106**, 8302-8307 (2009).

S100. A. Boronat *et al.*, Analysis of antibodies to surface epitopes of contactin-2 in multiple sclerosis, *J Neuroimmunol* **244**, 103-106 (2012).
